# Orthodontic management of a developing dentigerous cyst related to lower second molar: a case report

**DOI:** 10.1186/s12903-023-03731-3

**Published:** 2023-12-14

**Authors:** Amr Ragab El-Beialy, Abdulmonem Moftah BinRahima, Mohammed Al Shhab, Yehya Mostafa

**Affiliations:** 1 Department of Orthodontics and Dentofacial Orthopedics, Faculty of Dentistry, Cairo University, 11 El-Saraya Street, Manial, Cairo Egypt; 2 Cairo University, Giza, Egypt; 3 Future University in Egypt, New Cairo, Egypt; 4 Department of Orthodontics, Future University in Egypt, New Cairo, Egypt

**Keywords:** Dentigerous cyst, Decompression, Orthodontic traction

## Abstract

The dentigerous cyst is a developmental odontogenic asymptomatic cyst, that is associated with the crown of an unerupted or impacted tooth. Early diagnosis is important to avoid any future complications and choose the best treatment option. The purpose of this case report is to describe the management of a dentigerous cyst related to lower second molar in a young female patient using orthodontic traction as a conservative treatment approach. This procedure helps to spare the patient an unnecessary surgical excision procedure and the associated excessive bone removal for a safety margin, stimulates bone healing and promotes the eruption of the cyst-associated tooth.

## Introduction

The presence of an impacted, unerupted, embedded tooth, a tooth under development, odontome or a supernumerary tooth, carries the risk of development of one of the most common types of odontogenic cysts; the Dentigerous cyst or follicular cyst [1]. It was reported that this type of cyst can be related to most of the permanent teeth [2] as well as the deciduous dentition [3]. The third molars are most frequently involved, although there is a substantial incidence among second molars and mandibular premolars [4].

The dentigerous cyst surrounds the crown of the unerupted tooth and is attached to its cervical region. It develops from accumulation of fluid between the remnants of the reduced enamel epithelium and the subjacent tooth crown shortly after complete formation of the crown [5]. The nature of the Dentigerous cyst, being painless and asymptomatic is the main reason behind the detection of this cyst after it attains large sizes. The Dentigerous cyst is usually detected by routine radiographic examination, prior to orthodontic treatment, or during the diagnosis of a failed tooth eruption [6].

Radiographically, the Dentigerous cyst is usually characterized by a unilocular radiolucent lesion around the unerupted tooth, with a distinct dense periphery of condensed bone [7]. An enlarged follicular space is usually misdiagnosed for a Dentigerous cyst [6]. However when the radiographic image depicts space larger than 5 mm, it denotes a Dentigerous cyst [8].

The classical treatment decision of the Dentigerous cyst is total enucleation with a safety margin of bone removal, to avoid recurrence together with extraction of the associated impacted tooth [9]. However, when there is an opportunity of preservation and eruption of the tooth, marsupialization or decompression of the cyst via fenestration is elected as a conservative treatment [10].

This article presents a clinical case with a Dentigerous cyst surrounding the crown of an unerupted lower right second molar that has been treated by orthodontic traction of the unerupted tooth.

## Case report

**Fig. 1:**
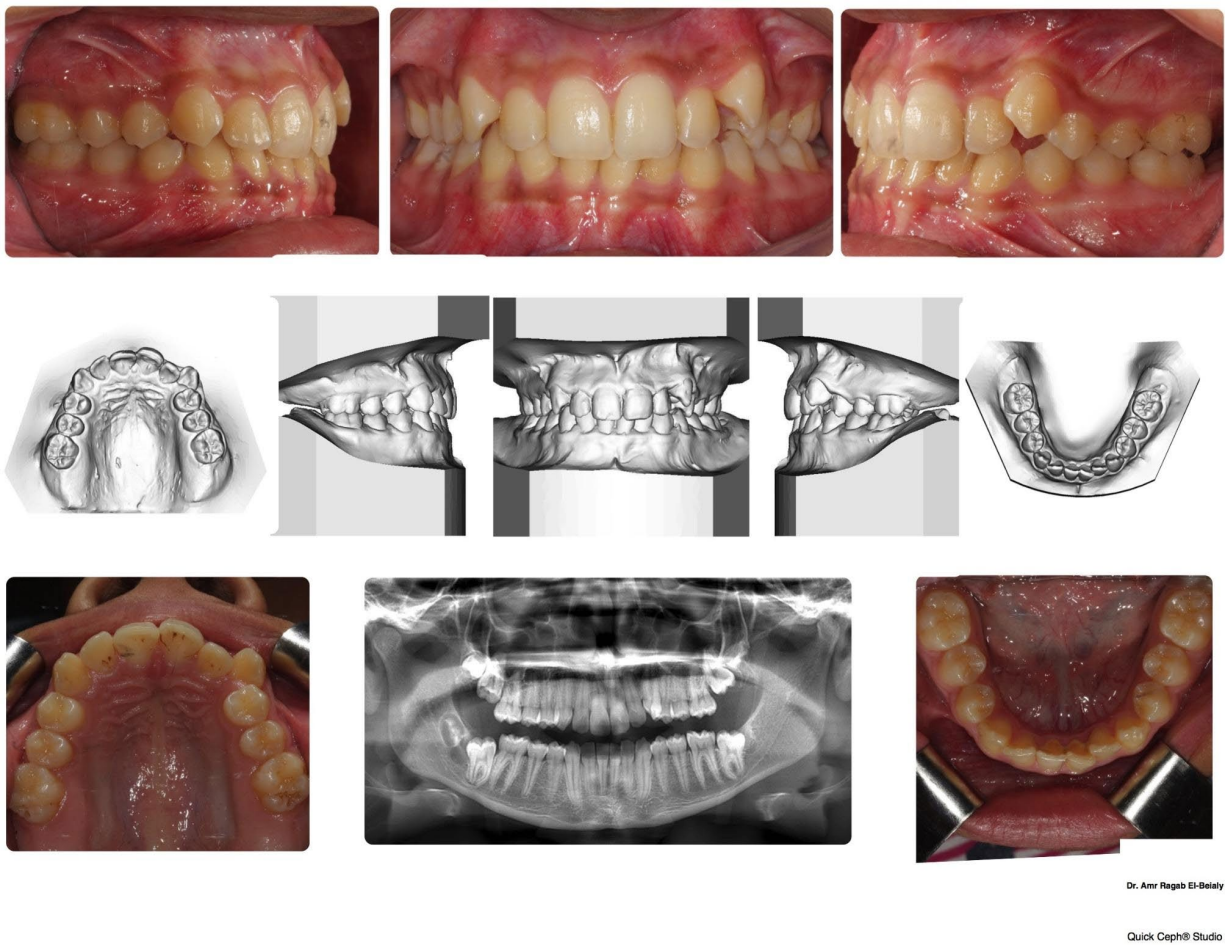
The pre-treatment records

A fourteen-year-old female presented to our orthodontic clinic with a chief complaint of unpleasant appearance of her teeth. The pre-treatment records including clinical intra-oral and extra-oral examination, photos, panoramic radiograph, lateral cephalometric radiograph, upper and lower impressions were taken. Intraoral examination showed the patient had a full set of erupted permanent teeth up to the first molars in all quadrants. A single cusp tip of the lower left second molar was erupting, while the lower right second molar was unerupted. The patient showed an Angle class I malocclusion with moderate and mild crowding in the upper and lower arches respectively, and anterior deep bite (Fig. 1). Extraoral examination showed a straight anterior divergent profile with over-competent lips. Examination of the panoramic radiograph (OPG) depicted a well-defined, circumscribed, unilocular, circular radiolucency surrounding the distal half of the crown of the unerupted lower right second molar with completely formed roots. The radiolucency is extending to the neck of the tooth, and which might be the cause of its impaction.

The case was referred to the maxillofacial surgeon for consultation. Based on the clinical, radiological and CBCT findings, a diagnosis of dentigerous cyst was concluded.

## Treatment

### Treatment option

The treatment option recommend by the surgeon was extraction of the associated impacted tooth and the third molar, cyst enucleation and bone removal with a proper safety margin in order to minimize the possibility of recurrence and allow the regeneration of healthy bone.

### Aims and objectives

**Fig. 2:**
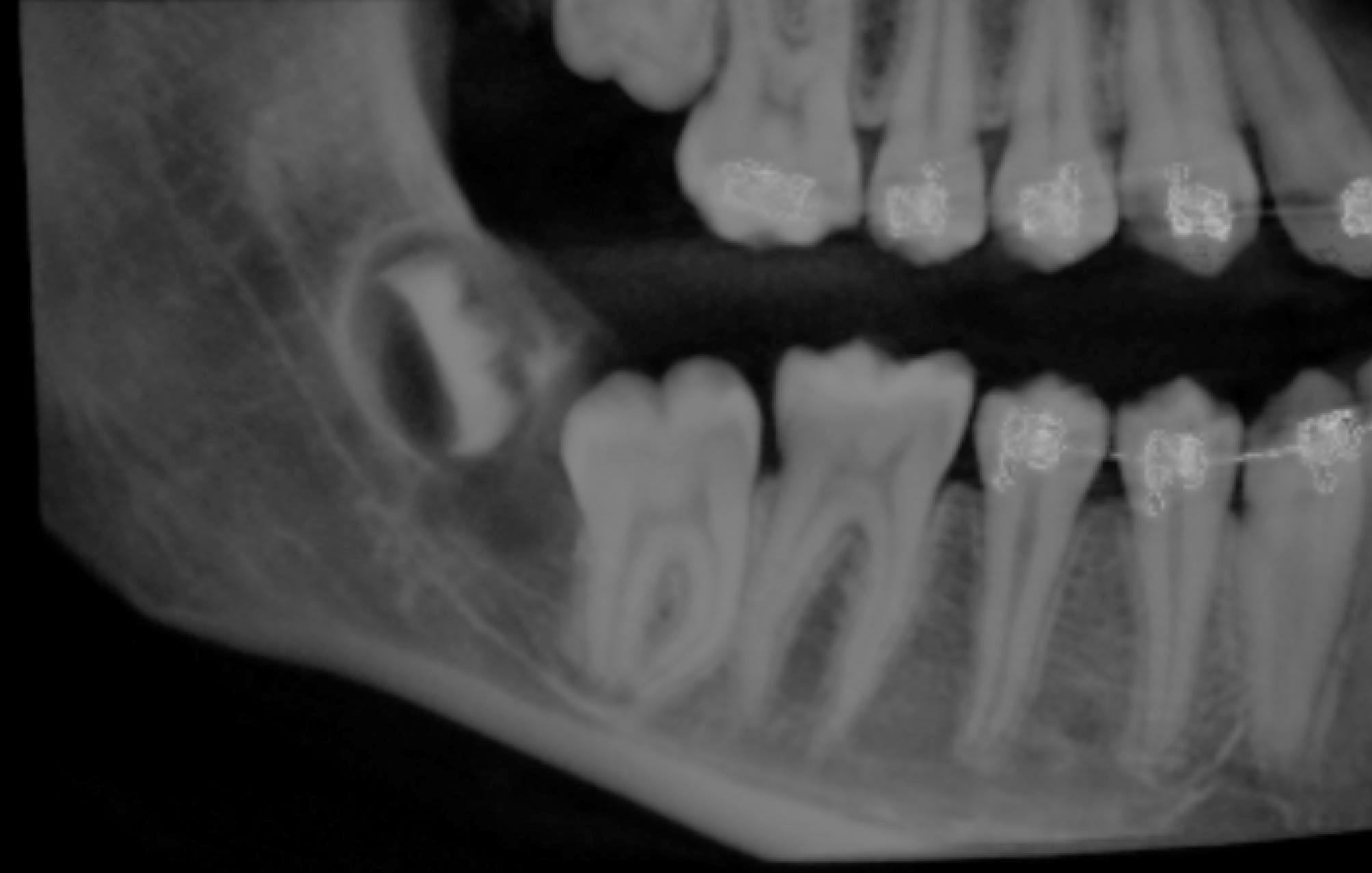
CBCT of the dentigerous cyst and the related second molar

Considering the age of the patient, size of the cyst, the position of the cyst close to the oral mucosa, the dental deep overbite, the possibility of resolution of the cyst with the eruption of the second molar, the approach of enucleation and tooth extraction was considered to be aggressive for the current case. Hence, a more conservative approach for the sake of conservation of the natural permanent tooth was agreed upon with the surgeon. Orthodontic traction which will be accompanied by spontaneous decompression and marsupialization of the cyst will be done. Accordingly, a CBCT was captured (Fig. 2), which confirmed the abovementioned diagnosis.

### Treatment plan

The treatment strategy depends on surgical exposure of the impacted second molar, bonding orthodontic attachment and orthodontic traction until the cyst lining attached at the cervical margin gets exposed in the oral cavity and blends with the oral mucosa, with no future risk of fluid accumulation and re-formation of the cyst. The critical stage in this procedure is the ability to maintain the decompression and cyst opening exposed in the oral cavity without healing until eruption of a part of the tooth.

### Treatment progress

**Fig. 3:**
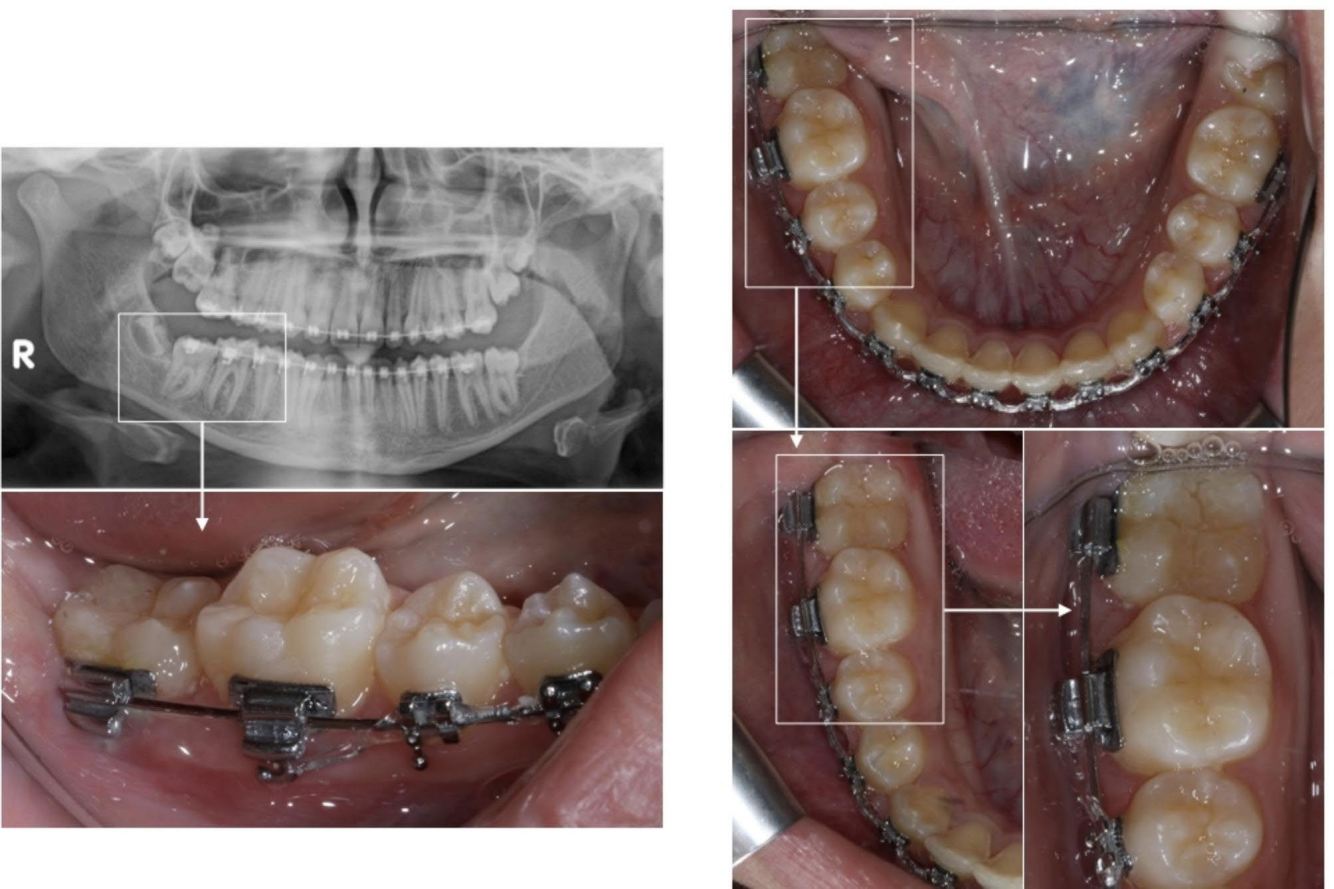
Records after eruption of the lower right second molar

Orthodontic treatment was initiated on the lower arch, comprising bonding of fixed orthodontic appliance, Roth prescription, 0.018 inch slot Roth brackets, with bonding of a double tube on the lower right first molar to help orthodontic traction of the lower second molar. Levelling and alignment was initiated until an archwire of 0.017 × 0.025 inch stainless steel archwire was reached. Surgical exposure of the second molar and bonding of an orthodontic tube to the exposed part of the buccal surface was done. An initial auxillary archwire 0.012 inch nickel titanium archwire attached between the auxillary tube of the first and second molars is used for occlusal traction of the second molar. In the monthly recall of the patient, the gauge of the wire was increased until the tooth is totally erupted on the oral cavity and an archwire of 0.017 × 0.025 inch stainless steel archwire could be inserted as a main archwire. At this stage, intraoral photographs and a panoramic radiograph were taken (Fig. 3), which shows no evidence of the Dentigerous cyst. The time taken from exposing the second molar until it got engaged into the main archwire was 4 months. Three years after treatment, a panoramic follow up radiograph was taken, and it showed no evidence of recurrence of the cyst.

## Discussion

The threat the dentigerous cyst presents is reverted back to its asymptomatic, incipient, and expanding nature. The early diagnosis is of paramount importance prior to the expression of the aggressive form of the lesion. This aggressive form has the potential to expand the bone of the mandible, with the subsequent displacement of teeth, malocclusion, pain, inferior alveolar nerve paresthesia and root resorption [5].

The intraoral observation of any altered eruption patterns, besides the proper examination of the pre-orthodontic panoramic radiograph to detect the presence of such cyst can be of significant help for early intervention. However, the differential diagnosis should be considered for identification of the Dentigerous cyst. When the follicular space exceeds 3–4 mm, Dentigerous cyst is suspected [8].

The most convenient treatment option for the dentigerous cyst should be chosen to suit the clinical situation. Surgical enucleation is a common modality for treatment of the Dentigerous cyst [11]. It encompasses complete removal of the cyst and cyst lining and the involved teeth and bone with a good safety margin to avoid recurrence.

The other treatment option is decompression; which is elected in the currently presented case and in other previously reported cases [12]. It encompasses cutting an incision into the cyst and maintaining this slit opened through suturing, for the sake of releasing the cystic fluid and avoiding the healing of the cystic wall. This will prevent re-accumulation of the cystic fluid and avoid any increase in intracystic pressure. The cyst must be marsupialized for decompression which can stimulate bone deposition to replace the space formerly occupied by the cyst and reduce the risk of damaging of adjacent structures [13]. This procedure releases the pressure on the bone and allows bone reformation and reduction in the size of the bony defect. This reduction in the size of the cyst preserves the involved teeth, decreases the amount of damage to the surrounding bone, [14] and decreases the possibility of recurrence.

In the current case, the young age of the patient with the strong healing and eruptive power of the teeth, besides the favorable position of the cyst and the vertical impaction of the second molar privileged decompression and orthodontic traction of the embedded second molar. Besides, the preservation of the second molar could have a favorable functional and psychological impact on the patient [15]. This procedure preserved the tooth associated with the cyst and facilitates its eruption. Similarly, ArifeKapdan [10] and Manoela Carrera [12] presented a case whereby the decompression technique was used. However, they extracted the preceding deciduous tooth to decompress the cyst, and awaited the spontaneous eruption of the permanent tooth related to the dentigerous cyst, unlike the case presented here where orthodontic traction is used to erupt the permanent tooth.

**Fig. 4:**
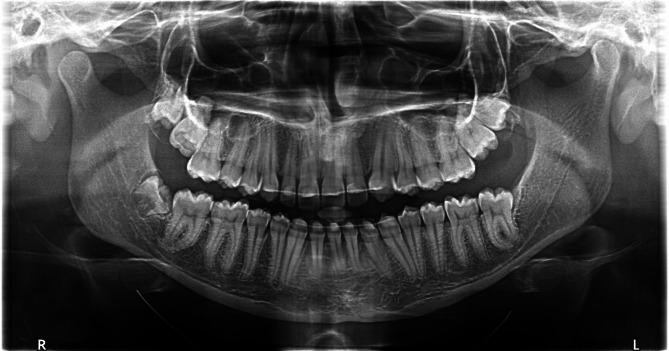
Panoramic radiograph 3 years after treatment

The bonding of the impacted second molar on the day of incising the cyst wall and decompression and the early occlusal traction of the second molar gave chance for maintaining the intact and the fast eruption of the impacted tooth. After few months of released pressure on the bone, the panoramic radiograph showed complete disappearance of the cyst, increased radio-opacity of the surrounding bone with the full eruption of the second molar. These same findings were confirmed in the panoramic radiograph taken 3 years after finishing the orthodontic treatment, with no evidence of recurrence (Fig. 4).

## Conclusion

The chosen treatment option of decompression of the developing dentigerous cyst simultaneously with guided eruption of the associated second molar showed acceptable results with such a case.The post-treatment panoramic radiograph showed complete radiographic healing of the cyst and uneventful eruption of the impacted second molar, which credits the treatment modality elected to save the involved tooth.

## Data Availability

All data generated or analysed during this study are included in this published article.
